# Purtscher-like retinopathy associated with dermatomyositis

**DOI:** 10.1186/1471-2415-13-36

**Published:** 2013-07-24

**Authors:** Yan Yan, Xi Shen

**Affiliations:** 1 Deparment of Ophthalmology, Ruijin Hospital Affiliated to Shanghai JiaoTong University School of Medicine, 197 Ruijin No.2 Road, Shanghai, 200025, China

## Abstract

**Background:**

To report a rare case of bilateral Purtscher-like retinopathy in a Chinese female patient with dermatomyositis.

**Case presentation:**

An 18-year-old woman with lower extremity muscle weakness was admitted due to decreased vision of both eyes for two weeks. Ophthalmic examination revealed violaceous eruption on eyelids with swelling. Ocular fundus examination revealed multiple cotton wool spots and Purtscher flecken, and intra-retinal haemorrhages with pseudo-cherry red spot. The erythrocyte sedimentation rate, C-reactive protein, lactate dehydrogenase and creatine kinase was measured and found to be increased. A diagnosis of Purtscher-like retinopathy associated with dermatomyositis was made and the patient was treated with corticosteroids.

**Conclusion:**

Although dermatomyositis is a rare cause of Purtscher-like retinopathy, it is important to keep in mind that systemic associations such as dermatomyositis should be ruled out in such patients.

## Background

Dermatomyositis is one of the idiopathic inflammatory myopathies. It is characterized clinically by progressive symmetrical proximal muscle weakness, a characteristic heliotropic rash on the eyelids and Gottron papules over bony prominences [1]. Retinopathy associated with dermatomyositis is rare and was first described by Bruce in 1938 [2]. Since then a few case reports have reported it in both adults and children [3]. The most predominant ocular lesion is a heliotropic rash. The characteristic retinal lesions include retinal hemorrhages and cotton-wool spots. Purtscher-like retinopathy is seen in patients with several kinds of connective tissue disorders, particularly systemic lupus erythematosis [4]. Acute fundus abnormalities in Purtscher-like retinopathy include Purtscher flecken, cotton-wool spots, retinal hemorrhage, and optic disc swelling [5]. To our knowledge, few reports have described Purtscher-like retinopathy associated with dermatomyositis [6]. Herein we report a rare case of bilateral Purtscher-like retinopathy in a Chinese female patient with dermatomyositis.

## Case presentation

**Figure 1:**
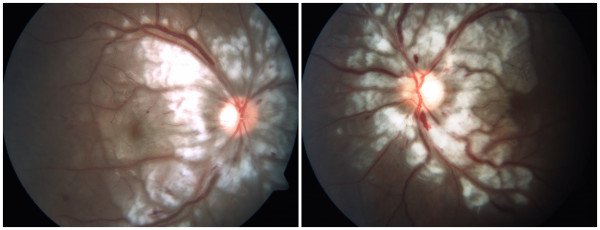
**An 18-year-old woman had decreased vision in both eyes for two weeks. **Fundus photographs show multiple cotton wool spots and Purtscher flecken with clear zone around the optic disc, intra-retinal haemorrhages and macular pseudo-cherry red spot.

**Figure 2:**
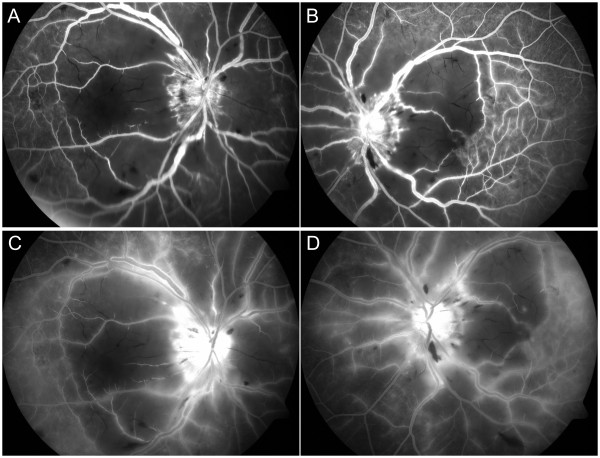
**Fluorescein angiography of a patient with dermatomyositis. A, B) **Fluorescein angiography shows retinal arterial and capillary occlusion around the optic disc and macular non-perfusion. **C, D) **Fluorescein angiography shows optic disc leakage, vessel wall staining of veins, venules and some arterioles in the late phase.

An 18-year-old Chinese woman presented to us with subacute painless decreased vision in both eyes for two weeks. She had recurrent urticarial rashes on her eye lids and dorsum of the hands two months ago. She also had arthralgia of the arms and legs with increasing fatigue since then. Her past medical history was unremarkable. On examination, she was found to have erythematous papules over the metacarpal and interphalangeal joints. Lower extremity muscle strength was reduced (grade 3 out of 5). She had difficulties in standing up from a squatting position. Ophthalmic examination revealed violaceous eruption on eyelids with swelling. Her best corrected visual acuity was 20/600 in both eyes. Slit-lamp examination of both eyes revealed no inflammation in anterior chamber and vitreous body. No RAPD was found. Intraocular pressure was within normal limits. Bilateral fundus examination showed bilateral multiple cotton wool spots and Purtscher flecken surround the optic disc, and a few intra-retinal haemorrhages with macular pseudo-cherry red spot (Figure 1). Intravenous fluorescein angiography revealed bilateral retinal arteriole and capillary occlusion around the optic disc and macular nonperfusion (Figure 2A, B), optic disc leakage,vessel wall staining of veins, venules and some arterioles in the late phase (Figure 2C, D). Bilateral Purtscher-like retinopathy was diagnosed. With the advice of a consultant dermatologist, laboratory examination was done and showed elevated levels of leucocytes and muscle enzymes, which included white blood cell count 13.8×10^9^/L, neutrophils 96.7%, platelet count 82×10^9^/L, mean platelet volume 8.7fl, hemoglobin level 88 g/L, creatinine kinase 341 U/L, lactate dehydrogenase 575 U/L, alanine aminotransferase 354 U/L and aspartate aminotransferase 77 U/L. The bone marrow examination and peripheral blood smear were normal. The erythrocyte sedimentation rate was 45 mm/hr. C-reactive protein was 36.2 mg/L. The rheumatoid factor, antinuclear antibody, anti-antibodies to extractable nuclear antigen, anti-neutrophil cytoplasmic antibody and anti-Jo1 antibody were all negative. Serum viral antibody tests and anti-toxoplasmosis antibody were negative and tumour markers were not raised. Thyroid function tests were normal. The chest X-ray was normal. The electromyography study revealed myopathic features. A diagnosis of bilateral Purtscher-like retinopathy associated with dermatomyositis was made. Subsequently she was referred to the dermatological department and began treatment with intravenous methylprednisolone. Vision loss progressed to 20/400 in both eyes over the next 3 months in spite of treatment with corticosteroids. Ocular examination revealed bilateral optic disc pallor, attenuated retinal arteries and mottled RPE.

Purtscher's retinopathy was first described in 1910 by Otmar Purtscher, in a patient with severe head trauma [7]. Since then, a similar retinal appearance has also been described in a variety of conditions including acute pancreatitis, crush injury, long bone fracture, orthopaedic surgery, childbirth, and connective tissue disorders [4]. This case was characterized by the occurrence of Purtscher-like retinopathy as an early complication of dermatomyositis. The diagnosis of dermatomyositis was according to the criteria of Bohan and Peter [8].

The pathognomonic Purtscher flecken occur in only 50% of cases [9], and they were poorly recognized and documented in many patients [5]. These polygonal areas of retinal whitening occur in the inner retina, between the affected retinal arterioles and venules and have a characteristic clear zone, which described as an average of 50 μm unaffected zone on either side of the retinal arteries and precapillary arterioles [4]. Large emboli produce the confluent retinal whitening, which can be seen in branch retinal arterial obstruction, whereas distal retinal capillaries occlusion by small emboli forms the cotton-wool spots. Purtscher flecken results from occlusion of precapillary arterioles by intermediate-sized emboli.

Several kinds of emboli have been reported to participate in the pathogenesis of Purtscher’s retinopathy and Purtscher-like retinopathy, such as air, fat, leukocyte aggregates, fibrin, platelets and complement activation [4]. Brigitte et al. have described Purtscher-like retinopathy in a 7-year-old boy with severe juvenile dermatomyositis, suggesting that retinal vascular involvement might result from thrombotic thrombocytopenic purpura (TTP) rather than as a complication of juvenile dermatomyositis [6]. However, dermatomyositis seems to have been the cause of our patient’s retinopathy because she didn’t meet the criteria for TTP. Dermatomyositis is characterized pathologically by varying degrees of perifascicular atrophy, vasculopathy, and perivascular inflammation [1]. Obviously some factors such as leukocytes and inflammation may play an important role in our patient. Leukocyte aggregates induced by complement activation, which occur in connective tissue disorders, are usually up to 50 μm in diameter and may occlude the 45 μm diameter precapillary arterioles in the human retina [4]. Vasculitis due to the capillary endothelial damage by inflammatory factors may also contribute to the pathogenesis.

At present no treatment has been proven to be definitely effective [5]. Isolated case reports suggest that treatment of Purtscher's retinopathy with high dose intravenous steroids may be beneficial [10]. While in a prospective observational study, spontaneous visual recovery of at least 2 Snellen lines is seen in half of the cases [6]. The poor outcome of visual acuity may be associated with the optic disc or macular involvement and the extent of retinal vascular occlusion [11].

## Conclusion

In summary, Purtscher-like retinopathy rarely presents as an early manifestation of dermatomyositis. To avoid missing the diagnosis, attention should be paid to identify the characteristic Purtscher flecken. A patient with Purtscher-like retinopathy should be carefully examined and have lab work performed to rule out dermatomyositis. Treatment with systemic steroids may improve visual outcome in some patients, but the visual prognosis is also influenced by the amount of emboli as well as its location and stability.

### Consent

Written informed consent was obtained from the patient for publication of this case report and any accompanying images. A copy of the written consent is available for review by the Editor-in-Chief of this journal.

## Competing interests

The authors declare that they have no competing interests.

## Authors’ contributions

The work presented here was carried out in collaboration between all authors. YY took and edited the images. Both authors read and approved the final manuscript.

## Pre-publication history

The pre-publication history for this paper can be accessed here:

http://www.biomedcentral.com/1471-2415/13/36/prepub

## References

[B1] Robinson AB Reed AM Clinical features, pathogenesis and treatment of juvenile and adult dermatomyositis Nat Rev Rheumatol 2011 7 11 664 675 10.1038/nrrheum.2011.139 21947177

[B2] Bruce G Retinitis in dermatomyositis Trans Am Ophthalmol Soc 1938 36 282 292 16693160 PMC1315753

[B3] Venkatesh P Bhaskar VM Keshavamurthy R Garg S Proliferative vascular retinopathy in polymyositis and dermatomyositis with scleroderma (overlap syndrome) Ocul Immunol Inflamm 2007 15 1 45 49 10.1080/09273940601147653 17365808

[B4] Agrawal A McKibbin MA Purtscher's And purtscher-like retinopathies: a review Surv Ophthalmol 2006 51 129 136 10.1016/j.survophthal.2005.12.003 16500213

[B5] Miguel AI Henriques F Azevedo LF Loureiro AJ Maberley DA Systematic review of Purtscher's and purtscher-like retinopathies Eye (Lond) 2013 27 1 1 13 10.1038/eye.2012.222 23174749 PMC3545384

[B6] Bader-Meunier B Monnet D Barnerias C Halphen I Lambot-Juhan K Chalumeau M Costedoat-Chalumeau N Ribeil JA Bodemer C Gherardi R Thrombotic microangiopathy and purtscher-like retinopathy as a rare presentation of juvenile dermatomyositis Pediatrics 2012 129 3 e821 824 10.1542/peds.2011-0338 22311994

[B7] Purtscher O Noch unbekannte befunde nach schädeltrauma Ber Dtsch Ophthalmol Ges 1910 36 294 301

[B8] Bohan A Peter JB Polymyositis and dermatomyositis N Engl J Med 1975 292 8 403 407 10.1056/NEJM197502202920807 1089199

[B9] Agrawal A McKibbin M Purtscher’s Retinopathy: epidemiology, clinical features and outcome Br J Ophthalmol 2007 91 1456 1459 10.1136/bjo.2007.117408 17556428 PMC2095457

[B10] Atabay C Kansu T Nurlu G Late visual recovery after intravenous methyl prednisolone treatment of Purtscher’s retinopathy Ann Ophthalmol 1993 25 330 333 8297068

[B11] Holak HM Holak S Prognostic factors for visual outcome in purtscher retinopathy Surv Ophthalmol 2007 52 117 119 10.1016/j.survophthal.2006.10.012 17212995

